# The Mediating Role of Hopelessness in the Relationship Between Social Media Addiction and Loneliness Among Adolescents

**DOI:** 10.1111/jcap.70024

**Published:** 2025-05-08

**Authors:** Mustafa Durmuş, Abdullah Sarman, Necmettin Çiftci, Yusuf Durmuş

**Affiliations:** ^1^ Department of Nursing Muş Alparslan University, Faculty of Health Sciences Muş Turkey; ^2^ Department of Pediatric Nursing Bingöl University, Faculty of Health Science Bingöl Turkey; ^3^ Malazgirt Vocational School Muş Alparslan University Muş Turkey

**Keywords:** adolescent, hopelessness, loneliness, nursing, social media addiction

## Abstract

**Purpose:**

This study aims to explore the mediating role of hopelessness in the relationship between social media addiction and loneliness among adolescents.

**Methods:**

This study was conducted using a descriptive cross‐sectional correlational design. The study population comprised high school students in a provincial center in eastern Turkey. Through cluster sampling, 946 adolescents were included. This study was conducted between May 2024 and Sep 2024. Data collection involved a personal information form, the Social Media Addiction Scale for Adolescents (SMAAS), the UCLA Loneliness Scale Short Form (ULS‐8), and the Beck Hopelessness Scale (BHS). Data analysis was conducted using SPSS 25, G*Power 3.1, and AMOS 24 software.

**Results:**

The mean scores were as follows: SMAAS (20.44 ± 6.96), ULS‐8 (16.14 ± 3.97), and BHS (7.25 ± 5.16). Significant positive correlations were found between the scales. The model describing the relationships among social media addiction, loneliness, and hopelessness was within acceptable limits and yielded significant results (*Χ* ^2^/df=2.978, RMSEA = 0.052, CFI = 0.962, GFI = 0.971, IFI = 0.912). Social media addiction significantly affected loneliness, and time spent on social media also had a significant impact on loneliness (*p* < 0.001).

**Conclusions:**

Adolescents were found to use social media extensively, mainly for communication with friends and family. Hopelessness and time spent on social media were identified as mediators in the relationship between social media addiction and loneliness. Findings suggest that reducing social media use may help mitigate loneliness and hopelessness. Mental health assessments should consider social media behavior, and educational programs should address its psychological impacts. Child and adolescent psychiatric nurses should be trained to recognize signs of loneliness and hopelessness in adolescents at an early stage.

## Summary

- Adolescents use social media intensively, mainly to communicate with friends and family.
- Hopelessness and social media time mediate the link between addiction and loneliness.
- Interventions should target hopelessness and social media use to reduce loneliness effects.

## 1 Introduction

Adolescence is a period of transition to competence characterized by physical differentiation, emotional development, behavioral changes, and mental transformations. Loneliness is a feeling that can be overcome as a result of the individual's trust in others and contact with those to whom he/she feels socially connected (Nowland et al. 2018). Loneliness is experienced at all stages of life, but it is particularly prevalent during adolescence. Research indicates that between 21% and 70% of adolescents report feelings of loneliness (Lodder et al. 2016; Qualter et al. 2015).

Social media use involves internet‐based networking that facilitates verbal and visual interactions with others (Carr and Hayes 2015). Adolescents spend a significant portion of their leisure time on online social media platforms, making it one of the most popular activities during this stage of life (Azhari et al. 2022). Among youth, 97% of all adolescents between the ages of 13 and 17 use at least one of the following seven social media platforms: YouTube (85% of adolescents), Instagram (72%), Snapchat (69%), Facebook (51%), Twitter (32%), Tumblr (9%) or Reddit (7%) (Pew Research Center 2018). Social media enables individual expression (Radovic et al. 2017) but, when used excessively, can negatively impact mental health (Raudsepp and Kais 2019), leading to loneliness, insomnia, fatigue, diminished interest and concentration, depressive moods, and stress (Aalbers et al. 2019; Azhari et al. 2022; Van Rooij et al. 2017). Adolescents are particularly vulnerable to the effects of social media (Victor et al. 2024), as evidenced by a study indicating that those experiencing loneliness tend to spend more time on these platforms in an attempt to alleviate negative feelings (Sarman and Tuncay 2023).

Loneliness and hopelessness are often experienced concurrently, particularly during adolescence, a period marked by emotional sensitivity and heightened social awareness (Sundqvist and Hemberg 2021). Excessive use of social media, while initially serving as a tool for social connection, may contribute to these negative emotional states (Naslund et al. 2020). Adolescents may turn to social media to alleviate loneliness; however, when their efforts to connect online are unsuccessful or unfulfilling, it may exacerbate feelings of hopelessness. Thus, these parameters are interrelated, and their interactions are critical to understanding adolescent mental health in the digital age (Bonsaksen et al. 2023).

Hopelessness is define as a negative emotion caused by the belief that everything will get worse, that there are no positive expectations in life, and that problems cannot be solved (Beck et al. 1974). Hopelessness is a common experience among adolescents (Kulik and Sądel 2013). In a study with adolescents, researchers found a positive correlation between loneliness and hopelessness, indicating that as feelings of loneliness intensify, so does the level of hopelessness (AKKUŞ ÇUTUK 2021).

Child and adolescent psychiatric nurses play a critical role in evaluating the impact of contemporary issues, such as social media addiction and loneliness, on children and adolescents, as well as raising awareness in these areas (Carpenter and Hubbard 2014). Identifying the risk factors associated with social media addiction and loneliness and developing individualized care plans to mitigate these risks are key responsibilities of nurses. They can organize psychoeducation programs for children and families to address hopelessness and promote awareness of its effects. Additionally, nurses provide counseling services to manage symptoms of social media addiction, loneliness, and hopelessness through early intervention programs. Enhancing individuals' social interactions through initiatives such as group therapy and social skills development activities also falls within the scope of their responsibilities (Spence 2003).

This study aimed to determine the mediating role of hopelessness in the relationship between social media addiction and loneliness among adolescents.

### 1.1 Hypotheses

There is a relationship between social media addiction and loneliness.

There is a relationship between social media addiction and hopelessness.

There is a relationship between loneliness and hopelessness.

There is a mediating role of hopelessness and time spent on social media in the relationship between social media addiction and loneliness.

## 2 Material and Methods

### 2.1 Study Design

This study was conducted using a descriptive cross‐sectional correlational design.

### 2.2 Population and Sample

The study's universe comprised high schools in a provincial center in Eastern Turkey. The city center contains 22 public high schools, which were categorized by school type using the cluster sampling method. Each categorized high school received a numerical designation. Then, one high school was selected from each cluster using simple random sampling method with proportional probability and the selection process was completed. The Cochran formula (*n*=Z^2^xpxq/e^2^) was employed in the study (Cochran 1997). The calculations indicated that a minimum sample size of 320 participants was necessary. The study was completed with 946 participants. A post hoc power analysis conducted using the G*Power 3.1 statistical software confirmed that the study achieved a 95% confidence level with a large effect size (Cohen 1992). This study was conducted between May 2024 and Sep 2024.

#### 2.2.1 Inclusion Criteria

Students aged 14–18 years who volunteered, actively used social media, and had no health conditions impeding questionnaire completion were included in the study. Several reasons are the selection of the 14–18 age range for this study. First, social media use is highly prevalent in this age group, and their access to digital platforms is substantial. Second, as individuals in this age range actively exhibit key characteristics of adolescence, the psychosocial effects of social media use are expected to be more pronounced. Finally, the measurement tools employed in the study have been validated specifically for use within this age group.

#### 2.2.2 Exclusion Criteria

Data from students who declined participation or submitted incomplete questionnaires and scale responses were excluded from the study.

### 2.3 Data Collection Tools

A personal information form, created by the researchers following a literature review, along with the Social Media Addiction Scale for Adolescents, UCLA Loneliness Scale Short Form (ULS‐8), and Beck Hopelessness Scale, were employed for data collection. The study's reporting adhered to the STROBE guidelines for observational studies in epidemiology (Vandenbroucke et al. 2007).

#### 2.3.1 Personal Information Form

The form developed from literature review others (Sarman and Çiftci 2024; Victor et al. 2024; Sarman and Tuncay 2023), includes questions on gender, age, class, family income level, and parents' education levels, among.

#### 2.3.2 Social Media Addiction Scale for Adolescents (SMAAS)

The Social Media Addiction Scale for Adolescents (SMAAS) was developed in accordance with the APA DSM‐5 criteria (Özgenel et al. 2019). The scale is rated on a 5‐point Likert scale, with the following designations: Never‐1, rarely‐2, sometimes‐3, very often‐4, always‐5. The scale does not include any items scored in reverse, and it comprises 9 items. A score can range from a minimum of 9 to a maximum of 45 points. The total score of the scale is calculated by summing the responses to all items. In the study of validity and reliability, which included adolescents aged 11–18, a higher total score corresponds to a higher level of social media addiction, whereas a lower total score indicates low addiction. In the Turkish validity and reliability of the study, the scale's Cronbach's alpha reliability coefficient was reported as 0.90 (Özgenel et al. 2019). In this study, Cronbach's alpha coefficient was determined to be 0.87.

#### 2.3.3 Short‐Form of the UCLA Loneliness Scale (ULS‐8)

Short‐Form of the UCLA Loneliness Scale (ULS‐8), developed by Hays and DiMatte in 1987 (Hays and DiMatteo 1987), was adapted into Turkish by Yıldız and Duy in 2014 for individuals aged 17–28 years (Yıldız and Duy 2014). This unidimensional scale comprises seven items and utilizes a four‐point Likert scale with options: never (1), rarely (2), sometimes (3), and always (4). The 5th item is reverse‐scored, and the overall loneliness score is calculated by summing the responses to all seven items. Short‐form of the UCLA Loneliness Scale (ULS‐8) has a scoring range from a minimum of seven to a maximum of 28 points. Lower scores suggest a reduced experience of loneliness, while higher scores indicate an increased intensity of loneliness (Yıldız and Duy 2014). In the Turkish adaptation of the scale, the Cronbach's alpha reliability coefficient was also determined to be 0.74 (Yıldız and Duy 2014). In this study, Cronbach's alpha value was reported as 0.88.

#### 2.3.4 Beck Hopelessness Scale (BHS)

The Beck Hopelessness Scale (BHS) was developed by Beck et al. (1974). The Turkish validity and reliability study was conducted by Durak (1994) with individuals aged 15–65 (Durak 1994). The Beck Hopelessness Scale is a self‐assessment tool comprising 20 items. It is applicable to children, adolescents, and adults. Each question is answered with ‘yes’ or ‘no’, and scores are assigned accordingly. The scale includes items that are scored in reverse; for instance: Questions 2, 4, 7, 9, 11, 12, 14, 16, 17, 18, and 20 are awarded 1 point for a ‘yes’ response. Questions 1, 3, 5, 6, 8, 10, 13, 15, and 19 are awarded 1 point for a ‘no’ response. The total score on the scale can range from a minimum of 0 to a maximum of 20. Scores ranging from 0 to 3 signify normal hopelessness, 4–8 indicate mild hopelessness, 9–14 suggest moderate hopelessness, and a score of 15 or higher denotes intense hopelessness (Küçükceran et al. 2024). A study utilizing the scale with high school students aged 14–19 years revealed that the Cronbach's alpha coefficient varied from 0.73 to 0.85 across the total and sub‐dimensions (AKKUŞ ÇUTUK 2021). In this study, Cronbach's alpha coefficient was determined to be 0.75.

### 2.4 Data Collection Process

Data collection was conducted using Google Forms due to its capability to organize data via templates or worksheets, either independently or together. School administrators, identified by the researchers, were interviewed to explain the study's purpose. Subsequently, the classes were briefed on the upcoming study. The survey link was distributed to adolescents through online platforms like WhatsApp, Telegram, or email. The Google Form included information on the study's purpose, participation requirements, and the need for parental consent, thus ensuring participants were well‐informed. It was emphasized that the study would commence only after obtaining consent from both parents and adolescents. Participants were briefed on the questionnaire sections and informed that it would take approximately 10–15 min to complete. They were also assured that they could withdraw from the study at any point without needing to provide a reason.

### 2.5 Data Analysis

Analyses were conducted using SPSS 25.0, AMOS V 24.0, and G*Power 3.1 statistical software. The normality of the data was assessed. Descriptive statistics were presented as number, percentage, mean, and standard deviation. Compliance of the data with normal distribution was evaluated through the values of Skewness and Kurtosis. It is stated that if the Skewness and Kurtosis values are between −1.5 and +1.5, the data can be considered normally distributed (Tabachnick et al. 2013). The independent sample *t*‐test was utilized for pairwise group comparisons, while ANOVA was employed for comparisons among three or more groups. Mediation analysis was carried out using R programming language version 4.1.3, applying the method developed by Andrew Hayes (Hayes 2009). A significance level of *p* < 0.05 was established for all tests.

### 2.6 Ethical Approval

Before initiating the study, the non‐interventional ethics committee of Bingöl University granted approval (06.03.2024‐E.148966). Subsequently, the Directorate of National Education provided institutional permission to conduct research in high schools (15.04.2024‐153059). All participating adolescents were informed about the study's purpose, and assured that data would be maintained confidentially, as detailed in the explanation text linked to the online data collection tool. Parental consent was secured, and only those adolescents who read and consented to the information text were permitted to participate in the study. The study's procedures were carried out in strict adherence to the Declaration of Helsinki principles. Data were safeguarded on a password‐protected computer, accessible solely to the research team.

## 3 Results

The analysis revealed that the participants were homogenous across all socio‐demographic characteristics (*p* > 0.05). Furthermore, a significant gender‐based disparity was observed in the mean total scores of the BHS. Statistically significant differences were also noted in the mean total scores across all scales concerning parental attitude, income level, academic achievement, peer communication, and the perception of having an adequate number of friends (*p* < 0.05; Table 1).

**Table 1:** Distribution of socio‐demographic characteristics of participants by mean scale scores (*n* = 946).

Variables	*n*	%	Test *X* ^*2*^ (p)	SMAAS $\overset{¯}{X}$± SD	ULS‐8 $\overset{¯}{X}$± SD	BHS $\overset{¯}{X}$± SD
**Age**						
Female	435	46.0	1.132 (0.769)	20.78 ± 6.84	16.21 ± 3.98	7.72 ± 5.20
Male	511	54.0		20.03 ± 7.10	16.07 ± 3.96	6.84 ± 5.09
Test (p)				*t* = 1.643 (0.101)	*t* = 0.520 (0.603)	*t* = 2.635 (0.009)
**Mother's education level**						
Illiterate^1^	192	20.3	2.021 (0.568)	19.03 ± 6.72	15.75 ± 3.99	7.25 ± 5.35
Primary school^2^	273	28.9		22.40 ± 6.72	16.87 ± 4.02	7.90 ± 5.19
Middle school^3^	230	24.3		20.26 ± 6.65	16.05 ± 3.79	6.86 ± 4.94
High school^4^	251	26.5		19.55 ± 7.26	15.71 ± 3.96	6.88 ± 5.19
Test (*p*)				*F* = 11.617 (0.000) 2 > 3 > 4 = 1^a^	*F* = 4.771 (0.003) 2 = 3 > 1 = 4^a^	*F* = 2.339 (0.072)
**Father's education level**						
Illiterate^1^	35	3.7	7.796 (0.051)	13.29 ± 4.47	16.46 ± 3.28	7.17 ± 6.00
Primary school^2^	195	20.6		19.88 ± 7.46	15.80 ± 3.85	6.97 ± 5.04
Middle school^3^	294	31.1		20.71 ± 6.64	16.16 ± 4.11	6.74 ± 4.50
High school^4^	422	44.6		21.29 ± 6.55	16.34 ± 4.02	7.68 ± 5.43
Test (*p*)				*F* = 15.837 (0.000) 4 > 3 > 2 > 1^a^	*F* = 1.156 (0.325)	*F* = 1.871 (0.133)
**Class**						
9th^1^	330	34.9	0.781 (0.854)	20.64 ± 7.14	16.46 ± 3.76	6.49 ± 5.05
10th^2^	264	27.9		20.29 ± 7.44	15.73 ± 4.16	7.07 ± 4.53
11th^3^	129	13.6		21.09 ± 6.15	16.43 ± 4.36	7.49 ± 5.37
12th^4^	223	23.6		19.93 ± 6.56	15.96 ± 3.76	7.90 ± 5.22
Test (p)				*F* = 0.901 (0.440)	*F* = 2.050 (0.105)	*F* = 3.495 (0.015) 4 = 3 = 2 > 1^a^
**Parental attitude**						
Understanding^1^	393	41.5	15.847 (0.058)	18.99 ± 6.43	15.18 ± 3.74	6.08 ± 4.37
Perfectionist^2^	144	15.2		18.30 ± 6.37	14.85 ± 3.85	6.45 ± 4.85
Overprotective^3^	166	17.5		21.48 ± 7.58	16.61 ± 4.16	7.36 ± 5.34
Repressive^4^	172	18.2		22.88 ± 6.66	18.13 ± 3.34	8.99 ± 5.65
Disinterested^5^	71	7.5		24.45 ± 6.45	18.08 ± 3.67	10.83 ± 5.44
Test (*p*)				*F* = 21.462 (0.000) 5 > 4 > 3 > 1 = 2^a^	*F* = 28.011 (0.000) 4 = 5 > 3 > 1 = 2^a^	*F* = 20.998 (0.000) 5 > 4 > 3 > 2 = 1^a^
**Income level**						
Good^1^	167	17.7	14.190 (0.814)	20.85 ± 6.57	16.71 ± 4.09	5.93 ± 4.76
Moderate^2^	649	68.6		20.61 ± 6.82	15.87 ± 3.86	7.31 ± 5.10
Bad^3^	130	13.7		19.03 ± 7.98	16.72 ± 4.23	8.63 ± 5.55
Test (*p*)				*F* = 3.162 (0.043) 1 = 2 > 3^a^	*F* = 4.557 (0.011) 3 = 1 > 2^a^	*F* = 10.316 (0.000) 3 > 2 > 1^a^
**Academic achievement**						
Good^1^	214	22.6	0.172 (0.917)	18.32 ± 6.92	15.33 ± 4.16	4.86 ± 4.29
Moderate^2^	617	65.2		20.47 ± 6.80	16.09 ± 3.85	7.65 ± 5.05
Bad^3^	115	12.2		24.22 ± 6.35	17.91 ± 3.67	9.50 ± 5.59
Test (p)				*F* = 28.374 (0.000) 3 > 2 > 1^a^	*F* = 16.526 (0.000) 3 > 2 > 1^a^	*F* = 38.768 (0.000) 3 > 2 > 1^a^
**Peer communication**						
Good^1^	392	41.4	0.266 (0.875)	18.82 ± 6.53	14.21 ± 3.51	5.72 ± 4.67
Moderate^2^	435	46.0		20.87 ± 6.88	16.90 ± 3.60	7.81 ± 4.97
Bad^3^	119	12.6		24.17 ± 7.05	19.69 ± 3.20	10.22 ± 5.66
Test (p)				*F* = 30.198 (0.000) 3 > 2 > 1^a^	*F* = 128.978 (0.000) 3 > 2 > 1^a^	43.065 (0.000) 3 > 2 > 1^a^
**Having an adequate number of friends**						
Yes^1^	533	56.3	1.302 (0.522)	19.01 ± 6.64	14.53 ± 3.56	6.17 ± 4.78
No^2^	230	24.3		23.02 ± 6.96	18.80 ± 3.45	9.07 ± 5.28
Undecided^3^	183	19.3		21.36 ± 6.82	17.46 ± 3.38	8.09 ± 5.27
Test (p)				*F* = 30.456 (0.000) 2 > 3 > 1^a^	*F* = 136.002 (0.000) 2 > 3 > 1^a^	*F* = 30.300 (0.000) 2 > 3 > 1^a^
**Purpose of use of social media**						
Spending leisure time	142	15.02	2.725 (0.687)	22.66 ± 3.17	18.13 ± 2.09	8.77 ± 2.76
Fun	160	16.92		20.60 ± 4.62	18.02 ± 6.16	7.66 ± 5.16
Communicating with friends/family (posting or following posts)	238	25.16		21.65 ± 3.45	17.96 ± 3.23	8.66 ± 3.45
Watch video	204	21.56		23.21 ± 3.12	18.55 ± 5.11	8.85 ± 5.77
Meeting people	202	21.34		20.16 ± 5.73	17.89 ± 2.65	7.96 ± 2.56
Test (*p*)				*F* = 29.751 (0.514)	*F* = 30.602 (0.498)	*F* = 26.111 (0.507)
				***X̄*** ± **SS**		
**Age**				15.70 ± 0.79		
**Time spent using social media (h/day)**				4.11 ± 1.43		

*Note: X̄:* Arithmetic mean, SD: Standard deviation, t: Student t test, F: ANOVA test.

Abbreviations: BHS, Beck Hopelessness Scale; SMAAS, Social Media Addiction Scale for Adolescents; ULS‐8, Short‐Form of the UCLA Loneliness Scale.

a Bonferroni test, *X* ^*2*^: Chi‐square test.

When the distribution of the scores obtained by the participants from all three scales was analyzed, it was determined that the mean score for the SMAAS was 20.44 ± 6.96, the mean score for the BHS was 7.25 ± 5.16, and the mean score for the ULS‐8 was 16.14 ± 3.97 (Table 2).

**Table 2:** Descriptive statistics: mean, minimum, maximum, and median values of scale scores.

Scales	$\overset{¯}{X}$± SD	Med.	Min.	Max.
**SMAAS**	20.44 ± 6.96	20	9	36
**ULS‐8**	16.14 ± 3.97	16	7	27
**BHS**	7.25 ± 5.16	6	0	20

*Note: X̄:* Arithmetic mean, SD: Standard deviation, Med: Median, Min.: Minimum, Max.: Maximum, SMAAS: Social Media Addiction Scale for Adolescents, ULS‐8: Short‐Form of the UCLA Loneliness Scale, BHS: Beck Hopelessness Scale.

When the relationship between the mean scale scores of the adolescents participating in the study was analyzed using Pearson's correlation coefficient, it was found that there was a statistically significant positive relationship between all scales (*p* < 0.05; Table 3).

**Table 3:** The relationship between the mean scores of the scale.

Scales	SMAAS	ULS‐8	BHS
**SMAAS**	—		
**ULS‐8**	0.310^a^	—	—
**BHS**	0.289^a^	0.354^a^	—

^a^ Significant relationship at 0.01 level (Pearson's correlation coefficient), SMAAS: Social Media Addiction Scale for Adolescents, ULS‐8: Short‐Form of the UCLA Loneliness Scale, BHS: Beck Hopelessness Scale.

**Figure 1:**
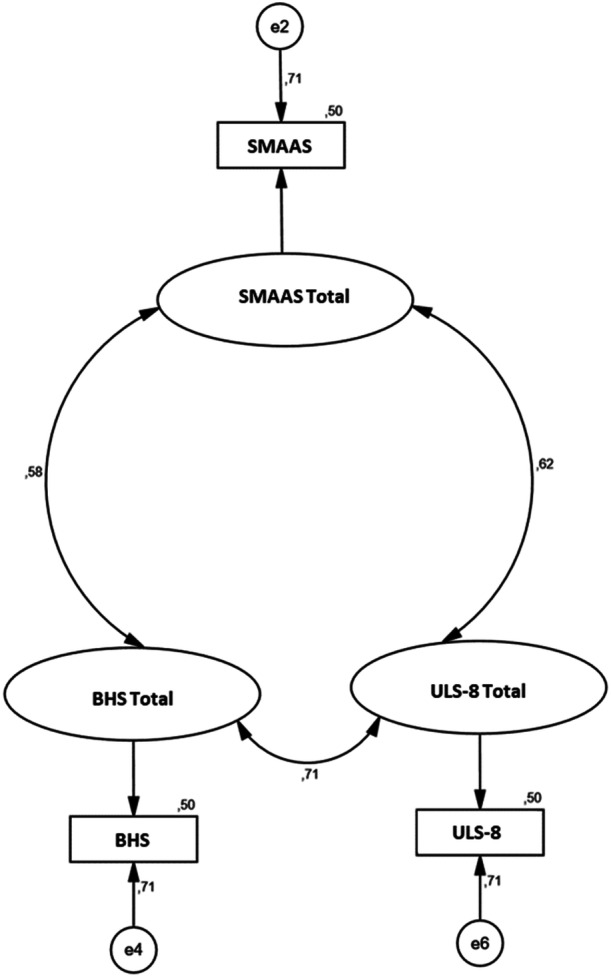
*Standardized Path Coefficients*.

A structural equation model (SEM) was established to determine the relationship between SMAAS, ULS‐8, and BHS. Upon examining the fit values, it was confirmed that the model was within the required limits, functioned correctly, and yielded significant results (*Χ* ^2^/df=2.978, RMSEA = 0.052, CFI = 0.962, GFI = 0.971, IFI = 0.912) (Table 4; Figure 1).

**Table 4:** Model fit indices.

			β^1^	β^2^	S.E.	C.R.	*p*
BHS	<‐‐>	ULS‐8	7.249	0.708	0.707	10.258	< 0.001
SMAAS	<‐‐>	BHS	10.381	0.578	1.217	8.532	< 0.001
SMAAS	<‐‐>	ULS‐8	8.560	0.619	0.942	9.091	< 0.001
	***Χ*** ^***2***^ **/df**	**RMSEA**	**CFI**	**GFI**	**IFI**	**AGFI**	**RFI**
	2.978	0.052	0.962	0.971	0.912	0.857	0.955

*Note:* β^1^: Standardized coefficients, β^2^: Non‐standardized coefficients, SE: Standard error, C.R.: Critical rate, *Χ* ^*2*^/df: Chi‐square/degrees of freedom, RMSEA: Root Mean Square Error of Approximation, CFI: Comparative Fit Indices, GFI: Goodness‐Of‐Fit Index, IFI: Incremental Fit Index, AGFI: Adjusted Goodness of Fit Index, RFI: Relative Fit Index.

**Figure 2:**
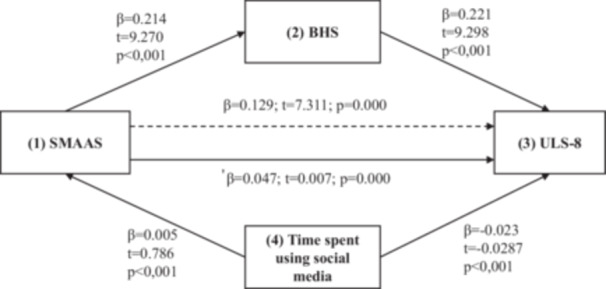
The Mediating Effect of Hopelessness and Time Spent Using Social Media on the Relationship Between Social Media Addiction and Loneliness. Statistical Model of Simple Mediation Analysis (Model 4). BHS: Beck Hopelessness Scale; SMAAS: Social Media Addiction Scale for Adolescents, ULS‐8: Short‐Form of the UCLA Loneliness Scale.

In this study, the mediating effect of hopelessness and time spent on social media on the relationship between social media addiction and loneliness was examined (Figure 2). For this purpose, direct regression analysis was performed between the variables and the results obtained are shown in Table 5. The data obtained showed that there was a relationship between the variables. These results were obtained using the bootstrap method with 5000 replications. According to the results, social media addiction was found to have an effect on loneliness (*β* = 0.214, *t* = 9.270, *p* = 0.000). It was seen that this condition explained 28% of loneliness. Similarly, hopelessness has a significant effect on loneliness (*β* = 0.221, *t* = 9.298, *p* = 0.000). This explains 17% of loneliness. Time spent on social media has a significant effect on loneliness (*β* = ‐0.023, *t* = 0.082, *p* = 0.000). It was determined that this situation explained 28% of loneliness.

**Table 5:** *simple mediation example (Model 4)*.

Variable	*β*	SE	*t*	*p*	LLCI	ULCI	*F*	R^2^
1–2 (direct effect)	0.214	0.023	9.270	< 0.001	0.168	0.259	85.949	0.288
1–3 (direct effect)	0.129	0.017	7.311	< 0.001	0.094	0.094	65.351	0.415
1–4 (direct effect)	0.005	0.006	0.786	< 0.001	−0.007	0.018	0.618	0.256
2–3 (direct effect)	0.221	0.023	9.298	< 0.001	0.175	0.268	5.766	0.172
3–4 (direct effect)	−0.023	0.082	−0.287	< 0.001	−0.185	0.138	13.097	0.288
**Total effect**	0.047	0.077	0.007	< 0.001	0.033	0.163		

*Note:* SE: Standard error, LLCI: Bootstrapping lower limit confidence interval, ULCI: Bootstrapping upper limit confidence interval, R^2^: Coefficient of determination.

## 4 Discussion

Today, social media is used almost everywhere (Azhari et al. 2022). With the integration of social media into various technological products, especially smartphones, the prevalence of use has increased. This situation resulted in 95% of the 15–24 age group turning to social media platforms (Tankovska 2021). It has been reported that the uncontrolled or excessive use of social media, particularly among adolescents, may lead to various problems (Verduyn et al. 2017). Social media addiction, a significant issue stemming from excessive use of social media, has been linked to a variety of mental health concerns (O'Day and Heimberg 2021).

This study revealed that adolescents dedicate a mean of 4.11 ± 1.43 h/day to time spent using social media. Furthermore, the mean total score for the SMAAS was 20.44 ± 6.96, while the ULS‐8's mean total score was 16.14 ± 3.97. A significant positive correlation was found between social media addiction and loneliness (*r* = 0.310; *p* < 0.01), indicating that higher social media addiction levels are associated with increased feelings of loneliness. In the study conducted by Bonsaksen et al. (2023), adolescents were found to spend a mean of 4.3 ± 1.4 h on social media daily, supporting these findings (Bonsaksen et al. 2023). This duration aligns with the results of other studies in the literature (Sarman and Çiftci 2024; Sarman and Tuncay 2023).

The relationship between social media use and loneliness remains paradoxical, as the literature presents mixed results. Sarman and Tuncay (2023) noted the lack of clarity surrounding this issue. Contrasting perspectives are evident, with Arampatzi et al. (2018) suggesting that while individuals may turn to social media to fulfill social needs, such usage could lead to increased feelings of loneliness. Meanwhile, Pittman (2015) reported that social media users are happier and experience less loneliness. Moreover, Sarman and Tuncay (2023) observed that spending excessive time on social media and addiction to it are linked to higher loneliness levels, while lonely individuals may also seek out social media as a coping mechanism. Wang et al. (2020) stated that those who follow others' posts on social media experience greater feelings of loneliness. The causal relationship between social media addiction and loneliness in adolescents remains uncertain due to a shortage of longitudinal research. It is hypothesized that adolescents might turn to the virtual world as a new habitat because one can easily spend considerable time on social media unwittingly, which may reduce real‐world interactions and thus increase loneliness. Further longitudinal studies are needed to clarify this relationship.

In addition, this study found a significant positive relationship between social media addiction and hopelessness (*r* = 0.289; *p* < 0.01). Adolescents were found to have a mild mean hopelessness score (7.25 ± 5.16). These findings suggest that adolescents with higher levels of social media addiction are more likely to experience feelings of hopelessness. Previous research supports this relationship: Leung (2014) found that concerns over online interactions and constant monitoring of social media feedback were linked to hopelessness among adolescents. Huang et al. (2023) also emphasized that adolescents use social media to fit in with their peers, while Victor et al. (2024) noted that disconnection from social media can contribute to feelings of exclusion and dependency. Although these results are consistent with existing literature, it was observed that social media addiction accounted for 28% of the mediating effect on hopelessness. This highlights the importance of investigating additional factors that contribute to social media addiction among adolescents. Another important finding is the positive relationship between loneliness and hopelessness (*r* = 0.354, *p* < 0.01). Students experiencing loneliness also showed higher levels of hopelessness, a result that echoes previous studies (Al Khatib 2012; Baran et al. 2015). When analyzed by gender, no significant difference was found in loneliness mean scores; however, females exhibited higher hopelessness scores than males (*p* < 0.05). The literature indicates that loneliness and hopelessness are often intertwined, particularly among female students who may face additional societal challenges such as gender discrimination and limited social opportunities in patriarchal cultures (Al Khatib 2012). In the region where the study was conducted, socioeconomic factors may also play a role. Lower socioeconomic status and reduced educational opportunities might contribute to increased feelings of hopelessness among adolescents. Baran et al. (2015) reported moderate to high levels of loneliness and hopelessness among Turkish adolescents, findings that align with the results of this study.

The results of this study confirm the interconnectedness of social media addiction, loneliness, and hopelessness among adolescents. It was found that adolescents experiencing loneliness are more prone to excessive social media use, which in turn can heighten feelings of hopelessness when virtual interactions do not yield meaningful connections. These findings align with recent studies (Huang et al. 2023; Victor et al. 2024) that emphasize how social media use may serve both as a coping mechanism and a risk factor, depending on the context and individual differences. Thus, addressing these factors in an integrated manner is essential for effective intervention. Moreover, social media addiction, hopelessness, and time spent on social media were found to mediate the relationship between social media usage and loneliness. Huang et al. (2023) reported a positive relationship between negative emotions and the risk of social media addiction, while Leung (2014) highlighted the mediating role of usage intensity. Victor et al. (2024) determined that individuals who frequently check social media and seek online validation are at greater risk of developing addiction. These findings suggest that adolescents experiencing hopelessness and loneliness may use social media more frequently, which, if uncontrolled, can escalate into addiction. Therefore, it is necessary to protect adolescents from the negative consequences associated with excessive social media use by implementing preventive measures. In this study, 25.16% of adolescents stated that their primary purpose for using social media was to communicate with friends and family. However, the purpose of social media use did not significantly affect any of the measured outcomes, consistent with findings by Bonsaksen et al. (2023).

Child and adolescent psychiatric nurses have a vital role in addressing these issues. They should educate families about setting boundaries for social media use, foster healthy digital habits, and help adolescents manage feelings of loneliness and hopelessness. Organizing community‐based educational programs and developing policy recommendations for digital health are essential strategies. In addition, child and adolescent psychiatric nurses can contribute to evidence‐based practices by engaging in research that explores new intervention models related to social media addiction, loneliness, and hopelessness. By integrating research findings into clinical practice, they can more effectively protect and promote the mental health of young populations.

### 4.1 Limitations and Strengths

The study's limitation to a single province and its cross‐sectional design prevents generalization and causal inference from the results. While a relationship between social media addiction and loneliness/hopelessness was observed, it cannot be concluded that prolonged social media use causes loneliness and hopelessness. A significant limitation is the lack of knowledge about participants' general behavior patterns and characteristics before the study. Additionally, levels of social media addiction, loneliness, and hopelessness in adolescents were assessed using self‐report scales. Another limitation is the absence of information on which social media platform(s) the adolescents use. Despite these limitations, the study's strengths include its large sample size, statistical power, use of structural equation modeling to analyze relationships among scale scores, and examination of hopelessness and social media use duration as mediators in the relationship between social media addiction and loneliness, which distinguish it from other studies.

### 4.2 Implications for Pediatric and Psychiatric Nurses

The results from this study on adolescents' social media usage have important implications for both pediatric and psychiatric nurses in practice.

### 4.3 Guidance on Social media Usage

Child and adolescent psychiatric nurses should take an active role in guiding adolescents toward healthier social media habits. Encouraging activities that promote emotional regulation and balance, such as sports, hobbies, and community involvement, is key. Child and adolescent psychiatric nurses can support adolescents in exploring interests outside the digital world, providing alternatives to excessive social media use that promote well‐being and personal development.

### 4.4 Family Education

Educating families is essential in fostering a supportive home environment that reduces dependence on social media. Child and adolescent psychiatric nurses should provide guidance to parents and caregivers on the importance of quality family time and open communication. By offering resources that enhance family bonding and engagement, nurses can help create environments where adolescents feel supported, reducing the reliance on social media for social interaction.

### 4.5 Recognition and Early Identification

Nurses need to be trained to recognize early signs of emotional difficulties in adolescents, such as loneliness and hopelessness. By being vigilant, pediatric and psychiatric nurses can identify at‐risk adolescents and intervene early to prevent the escalation of emotional and psychological issues. This proactive approach is critical in safeguarding adolescent mental health.

### 4.6 Adolescent Education

Educating adolescents about the potential risks of excessive social media use is another vital role for nurses. Pediatric nurses, in particular, can organize workshops and sessions to raise awareness among adolescents about social media addiction and its emotional impact. Providing adolescents with the knowledge and skills to manage their social media use responsibly can foster healthier online behavior and better emotional resilience.

### 4.7 Therapeutic Support

Collaboration with mental health professionals to provide therapeutic support is essential. Nurses can facilitate access to individual or group therapy for adolescents experiencing emotional challenges related to social media use, such as loneliness or hopelessness. By establishing a supportive therapeutic network, nurses can contribute to the mental well‐being of adolescents and help them navigate emotional difficulties more effectively.

By implementing these strategies, pediatric and psychiatric nurses can mitigate the negative effects of social media on adolescents, fostering a healthier, more balanced environment that supports both mental and emotional well‐being.

## 5 Conclusions

This study observed that adolescents frequently use social media throughout the day, primarily for communication with friends and family. The findings revealed a significant relationship between social media addiction and feelings of loneliness and hopelessness. Additionally, a positive correlation was identified between loneliness and hopelessness. The results further indicated that hopelessness and the amount of time spent on social media served as mediators in the relationship between social media addiction and loneliness.

Child and adolescent psychiatric nurses play a crucial role in guiding adolescents toward activities that help regulate social media usage, such as promoting social interaction and supporting their individual interests. Families should be educated on the importance of spending quality time with their children and improving communication within the family. Nurses should also be trained to identify early signs of loneliness and hopelessness in adolescents and intervene promptly.

Moreover, adolescents should receive education on the risks of social media addiction and appropriate social media use. By offering these trainings, child and adolescent psychiatric nurses can help raise awareness and foster healthier behaviors. Individual or group therapy sessions can also be organized to support adolescents in managing emotional challenges, such as loneliness and hopelessness. Collaborating with mental health professionals, nurses can provide adolescents with the tools to effectively cope with these difficulties.

## Author Contributions

**Mustafa Durmuş:** conceptualization, methodology, formal analysis, data curation, writing – original draft, writing – review and editing, visualization. **Abdullah Sarman:** conceptualization, methodology, formal analysis, data curation, writing – original draft, writing – review and editing, visualization. **Necmettin Çiftci:** conceptualization, methodology, formal analysis, data curation, writing – original draft, writing – review and editing, visualization. **Yusuf Durmuş:** conceptualization, methodology, formal analysis, data curation, writing – original draft, writing – review and editing, visualization.

## Consent

The adolescents were informed about the purpose of the study. Informed consent has been approved by all participants.

## Conflicts of Interest

The authors declare no conflicts of interest.

## Data Availability

The datasets used and/or analysed during the current study are available from the corresponding author on reasonable request.
